# Codelivery of Anticancer Drug and Photosensitizer by PEGylated Graphene Oxide and Cell Penetrating Peptide Enhanced Tumor-Suppressing Effect on Osteosarcoma

**DOI:** 10.3389/fmolb.2020.618896

**Published:** 2021-03-31

**Authors:** Yi-Fei Zhang, Yun-Feng Wu, Tai-Jin Lan, Yao Chen, Shi-Hong Su

**Affiliations:** ^1^ Department of Human Anatomy, West China School of Basic Medicine & Forensic Medicine, Sichuan University, Chengdu, China; ^2^ Department of Orthopaedics, The Fourth Affiliated Hospital of AnHui Medical University, Hefei, China; ^3^ Department of Respiratory and Critical Care Medicine, The First Affiliated Hospital of AnHui Medical University, Hefei, China

**Keywords:** graphene oxide, cell penetrating peptide, epirubicin, photodynamic therapy, osteosarcoma

## Abstract

**Objective:** Graphene oxide (GO) has been widely used for various biological and biomedical applications due to its unique physiochemical properties. This study aimed to investigate the effects of cell penetrating peptide (CPP) modified and polyethylene-glycol- (PEG-) grafted GO (pGO) loaded with photosensitive agent 2-(1-hexyloxyethyl)-2-devinyl pyropheophorbide-alpha (HPPH) and Epirubicin (EPI) (HPPH/EPI/CPP-pGO) on tumor growth in osteosarcoma.

**Methods:** The HPPH/EPI/CPP-pGO were prepared, and then in vitro drug release assay was conducted. The detection of singlet oxygen (^1^O_2_) and cellular uptake of HPPH was performed as well. Next, the effects of control (saline solution), CPP-pGO, EPI, HPPH, HPPH/CPP-pGO, EPI/CPP-pGO, HPPH/EPI/pGO, and HPPH/EPI/CPP-pGO were evaluated by MTT assay, colony-forming assay, and cell apoptosis assay in MG-63 cells. Furthermore, the antitumor effects of HPPH/EPI/CPP-pGO on osteosarcoma xenograft mice were unraveled.

**Results:** The ^1^O_2_ generation and cellular uptake of HPPH were significantly increased after CPP and pGO modification compared with free HPPH. In addition, compared with control cells, CPP-pGO treatment had low cytotoxicity in MG-63 cells. Compared with free HPPH or EPI, HPPH/CPP-pGO or EPI/CPP-pGO treatment significantly inhibited cell viability and colony forming number, as well as inducing cell apoptosis. HPPH/EPI-pGO treatment showed stronger inhibition effects on MG-63 cells than HPPH/CPP-pGO or EPI/CPP-pGO, and HPPH/EPI/CPP-pGO was the most effective one. Similarly, *in vivo* experiments revealed that, compared with control group, the tumor size and weight of osteosarcoma xenograft mice were obviously decreased after free HPPH or EPI treatment, which were further reduced in other groups, especially in HPPH/EPI/CPP-pGO group.

**Conclusion:** HPPH/EPI/CPP-pGO had superior tumor-inhibiting effects *in vitro* and *in vivo* on osteosarcoma.

## Introduction

Osteosarcoma is a primary bone malignant tumor originating in mesenchymal tissue in children and adolescents, characterized by high rates of invasiveness and mortality (Ottaviani and Jaffe, 2009). Sophisticated surgical resection combined with advanced chemotherapy and photodynamic therapy (PDT) is considered as the main and effective therapeutic methods for osteosarcoma (Elkordy et al., 2018; Harrison et al., 2017). Although the diagnosis and treatments for osteosarcoma have made intensive progress, delayed or missed diagnosis still results in unsatisfactory prognosis (Elkordy et al., 2018).

Epirubicin (EPI) as a highly effective broad-spectrum anticancer drug is usually used alone or in combination with other antitumor methods to exhibit powerful therapeutic effect for solid tumors, including osteosarcoma (Plosker and Faulds, 1993; Yu et al., 2019). However, due to the poor specificity and the insensitivity of mesenchymal tissue tumors to chemotherapeutic drugs, the clinical use of EPI is compromised (Plosker and Faulds, 1993).

In recent years, as the thinnest nanomaterial, graphene oxide (GO) has attracted more attention because of exceptional physical and chemical properties, including excellent biocompatibility and good thermal stability (Joshi et al., 2015). Recent studies have reported the biomedical applications of GO, including drug delivery, biomedicine, and cancer diagnosis (Kovbasyuk and Mokhir, 2016). Previous studies have demonstrated the antitumor effects of GO by delivering targeted chemotherapy drugs (Hwang et al., 2017; Rosli et al., 2019). In addition to GO, cell penetrating peptides (CPPs) are low molecular weight peptides with remarkable capacity for membrane translocation and can carry various macromolecules into cells including peptides, proteins, and nucleic acids (Ramsey and Flynn, 2015). As an effective transport tool, CPPs have successfully introduced a variety of cytotoxic drugs into tumor cells to induce apoptosis (Cao et al., 2018; Zhang et al., 2016).

PDT is a noninvasive treatment method which has been widely applied for cancers (Fink et al., 2015; Düzgüneş et al., 2018). PDT utilizes reactive oxygen species (ROS), such as mono oxygen or free radicals produced by irradiated photosensitization with appropriate wavelengths of visible or near-infrared spectra. ROS can oxidize various organelles including mitochondria, lysosomes, and nuclear membranes, ultimately leading to irreversible tumor cell damage (Stock et al., 2018). Unfortunately, the clinical application of PDT is limited by the hydrophobicity of many irradiation photosensitizers (Ozog et al., 2016).

In the current research, polyethylene-glycol- (PEG-) grafted GO (pGO) loaded with photosensitive agent 2-(1-hexyloxyethyl)-2-devinyl pyropheophorbide-alpha (HPPH) and EPI was used to enhance drug delivery to tumor cells through modification by CPP. The effects of HPPH/EPI/CPP-pGO on cell proliferation and apoptosis were investigated in MG-63 cells. Furthermore, the antitumor effects of HPPH/EPI/CPP-pGO on osteosarcoma xenograft mice were unraveled.

## Materials and Methods

### Preparation of GO-PEG (pGO), CPP-pGO, and HPPH/EPI/CPP-pGO

GO nanosheets were obtained from XFNANO Materials (Nanjing, China). The morphology and ultraviolet (UV) visible spectrum of GO were measured by the atomic force microscopy (AFM) and UV visible spectroscopy. First, pGO was generated by the modification of PEG on the surface of GO. Briefly, 1 mg/mL of GO was added into distilled water containing 5 mM of N-hydroxysulfosuccinimide sodium and 5 mM of N-(3-dimethylaminopropyl-N0-ethylcarbodiimide) hydrochloride and agitated at room temperature for 24 h to obtain the carboxyl-GO. Then, carboxyl-GO was mixed with 5 mM of NH2-PEG by stirring at room temperature for 24 h, and dialysis was performed to remove the excess salts and unreacted NH2-PEG. Next, to synthesize CPP-pGO, 100 μl of CPP (1 mM) and 10 mL of pGO (1 mg/mL) were mixed and stirred for 20 h at 4°C. After centrifugation, precipitates were harvested and CPP-pGO was obtained by freeze-drying in a vacuum dryer. Subsequently, CPP-pGO nanosheets were modified with HPPH and EPI. In brief, 2 mL of CPP-pGO (1 mg/mL) was mixed with 10 mM of HPPH that dissolved in dimethyl sulfoxide (DMSO) by stirring overnight, and dialysis was used to eliminate the redundant DMSO. After centrifugation for 10 min at 1600 g, the unloaded HPPH was also eliminated. Afterwards, 0.2 mg of EPI was added to either 1 mL of pGO (1 mg/mL) to produce EPI-loaded CPP-pGO (EPI/CPP-pGO), 1 mL of HPPH-complexed pGO (1 mg/mL) to produce EPI-loaded HPPH/pGO (HPPH/EPI/pGO), or 1 mL of HPPH-complexed CPP-pGO nanosheets (1 mg/mL) to produce HPPH/EPI/CPP-pGO nanosheets.

### In Vitro Drug Release Analysis

The drug release profiles of HPPH/EPI/CPP-pGO were evaluated by the dialysis bag method. The HPPH/EPI/CPP-pGO nanoparticle solution was dispersed in dialysis bag containing 1 mL of PBS and 0.1% Tween 80 (pH 7.4, 6.0, or 5.0). Then, the dialysis bag was immersed in 30 mL of the corresponding release medium and shaken at 100 rpm at 37°C. Subsequently, dialysate was withdrawn at different time. Finally, the concentrations of HPPH and EPI in the dialysate were respectively analyzed by fluorescence spectrophotometer and UV/Vis spectrometry.

### Cell Culture and Treatment

Human osteosarcoma cell line MG-63 was cultured in DMEM medium (Gibco). To evaluate the effects of HPPH/EPI/CPP-pGO on MG-63 cells, MG-63 cells were exposed to PBS (control), CPP-pGO, EPI (10 μg/mL), HPPH (1 μM), HPPH/CPP-pGO (containing 1 μM of HPPH), EPI/CPP-pGO (containing 10 μg/mL of EPI), HPPH/EPI/pGO (containing 1 μM of HPPH and 10 μg/mL of EPI), and HPPH/EPI/CPP-pGO (containing 1 μM of HPPH and 10 μg/mL of EPI), respectively, for 3 h. Next, the cells were irradiated for 5 min at 671 nm laser (2–8 mW/cm^2^) and incubated for 24 h.

### Cell Viability Assay

MG-63 cells underwent the above treatments and then were grown in 96-well plates for 24, 48, and 72 h. Next, 100 μL of CCK8 was added. After 1 h, the absorbances at 450 nm were obtained using microplate spectrophotometer.

### Colony-Forming Assay

400 cells/well of MG-63 cells were seeded in 6-well plates and then underwent the above treatments for 14 days. After being fixed with absolute methanol, crystal violet was used to stain cells. The cell number was calculated under light microscope.

### Cell Apoptosis Assay

MG-63 cells were exposed to different treatments for 24 h and harvested by trypsin. After being rinsed with PBS, cells were resuspended with buffer, followed by the exposure of FITC-Annexin V and PI. Cell apoptosis was observed using flow cytometer (BD, CA, United States).

### Detection of Singlet Oxygen

MG-63 cells were exposed to HPPH (1 μM) and HPPH/CPP-pGO (containing 1 μM of HPPH) and then mixed with 1.0 μM of singlet oxygen sensor green (SOSG). Cells were irradiated for 5 min at 671 nm laser (75 mW/cm^2^) and the singlet oxygen (^1^O_2_) level was detected by observing SOSG fluorescence at 494 nm under confocal fluorescence microscope.

### Cell Uptake Assay

Briefly, MG-63 cells were exposed to HPPH (1 μM), HPPH/pGO (containing 1 μM of HPPH), and HPPH/CPP-pGO (containing 1 μM of HPPH), respectively, for 24 h. After being washed with PBS, cells were fixed with 4% paraformaldehyde solution and permeabilized with Triton X-100. DAPI was used for the staining of nucleus. The excitation and emission at 425 and 725 wavelengths were used for HPPH imaging using confocal fluorescence microscope.

### Animal Model and Treatments

This study was approved by the Ethics Committee of our hospital. Healthy nude mice (18–22 g) were purchased and used for the experiments. To obtain the mouse xenograft model, 1 × 10^6^ of MG-63 cells per mouse were subcutaneously inoculated. Then, xenograft mice were treated with saline solution (control group, *n* = 4), HPPH/CPP-pGO (containing 1 mg/kg of HPPH, *n* = 4), EPI/CPP-pGO (containing 5 mg/kg of EPI, *n* = 4), HPPH/EPI/pGO (containing 1 mg/kg of HPPH and 5 mg/kg of EPI, *n* = 4), or HPPH/EPI/CPP-pGO (containing 1 mg/kg of HPPH and 5 mg/kg of EPI, *n* = 4) via rapid tail vein injection every other day. Twenty-four hours after every administration, mice received laser irradiation for 0.5 h at 671 nm laser (2–8 mW/cm^2^). After 3 weeks, mice were treated with Adriamycin. The tumor volume and body weight of mice were observed. On day 30, the tumor from mice was obtained to calculate tumor weight.

### Drug Biodistribution

For biodistribution analysis, the mice were killed after 3 weeks of treatment and the tumors and organs (spleen, kidneys, liver, lung, and heart) were collected and immediately frozen for cryosectioning at a 7 μm thickness. After staining the cell nuclei in the sections with DAPI, fluorescence images of tumors and tissues were obtained using a confocal scanning laser microscope. The relative fluorescent intensity was quantified and normalized to the control group.

### Serum Biochemical Analysis

After 3 weeks of treatment, serum samples were collected to analyze the levels of albumin (Alb), alkaline phosphatase (ALP), aminotransferase (AST), alanine aspartate aminotransferase (AST), total bilirubin (T-Bili), blood urea nitrogen (BUN), and the creatinine (Crea).

### Immunohistochemistry

Tumor tissues were treated with formalin-fixation, paraffin embedding, and slice preparation. After deparaffinization and dehydration, the sections were treated with citrate buffer (pH 6.0), followed by the heat pretreatment at 80°C and blocking with endogenous peroxide. Next, the sections were incubated with Ki-67 antibody, followed by the incubation of secondary antibody. Ultimately, the sections were mounted with neutral resin and observed under a light microscope (Nikon, ECLIPSE CI). The collected images were analyzed using Image-Pro Plus 6.0 software (Media Cybernetics, Inc., Rockville, MD, United States).

### Statistical Analysis

Data were expressed as the mean ± SD. Data comparison was performed using one-way ANOVA followed by multiple comparison using SPSS software. *P* < 0.05 was considered statistically significance.

## Results

### Drug Characterization and In Vitro Drug Release of HPPH/EPI/CPP-pGO

**FIGURE 1:**
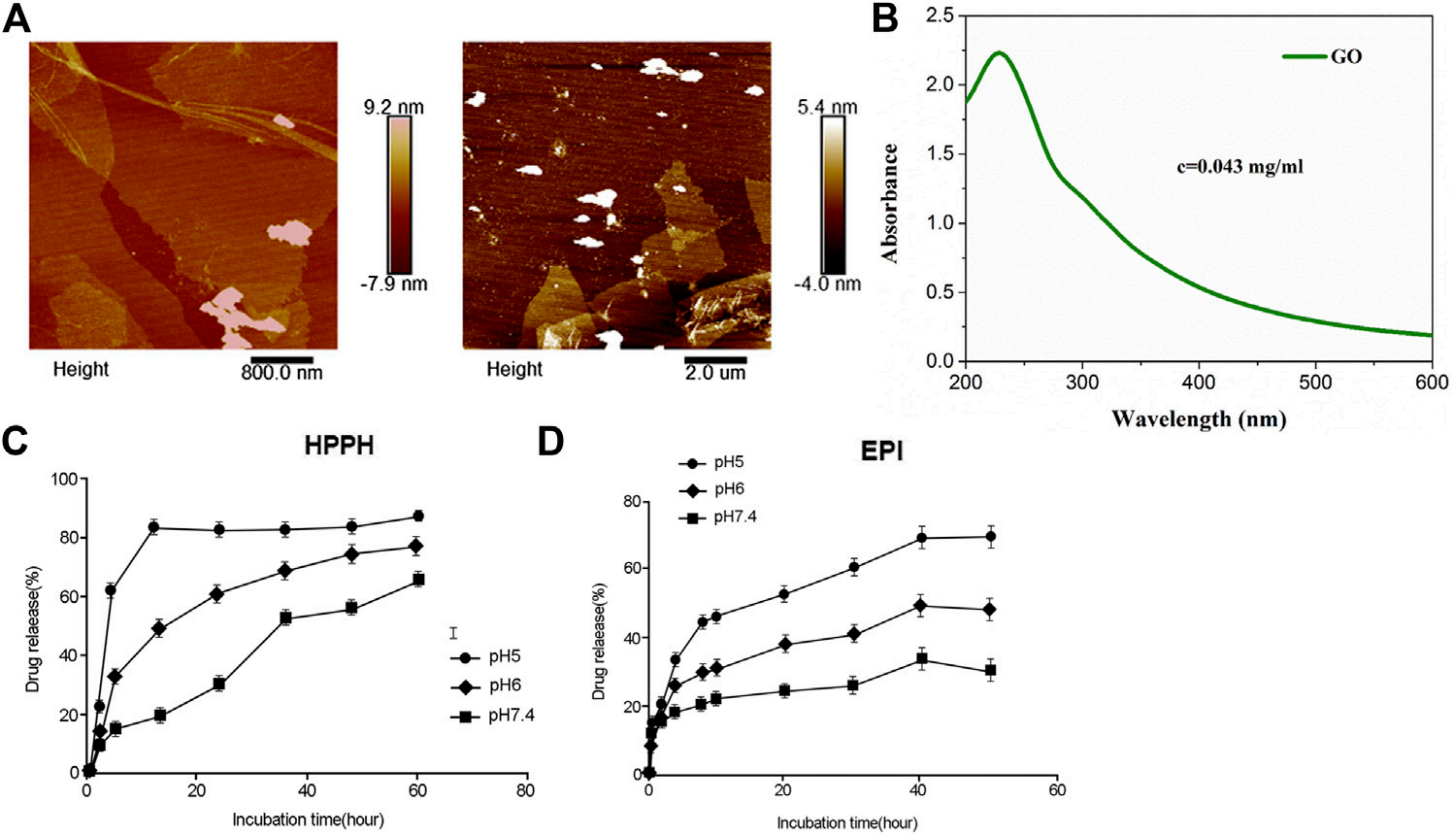
Characterization and in vitro drug release of HPPH/EPI/CPP-pGO. **(A)** The representative images of GO using atomic force microscopy. **(B)** The ultraviolet visible absorption spectra of GO. **(C)** Cumulative HPPH release profiling of HPPH/EPI/CPP-pGO in phosphate-buffer saline (PBS, pH 7.4, 6.0, and 5.0). **(D)** Cumulative EPI release profiling of HPPH/EPI/CPP-pGO in phosphate-buffer saline (PBS, pH 7.4, 6.0, and 5.0).

As determined by AFM, GO showed 100–400 nm of diameter and 4–5 nm of thickness (Figure 1A). An absorption peak at 230 nm was determined for GO (Figure 1B). Next, the amounts of HPPH and EPI released from HPPH/EPI/CPP-pGO were examined. The results revealed that HPPH was quickly released from HPPH/EPI/CPP-pGO within 5 h at pH 7.4 to pH 5.0 (Figure 1C), and about 90% of HPPH was released within 24 h at pH 5.0 (Figure 1C). In addition, HPPH/EPI/CPP-pGO exhibited a gradually increased release of EPI from 0 h to 50 h (Figure 1D), and almost 70% of EPI was released from HPPH/EPI/CPP-pGO within 50 h at pH 5.0 (Figure 1D).

### ^1^O_2_ generation and Cellular Uptake of HPPH

**FIGURE 2:**
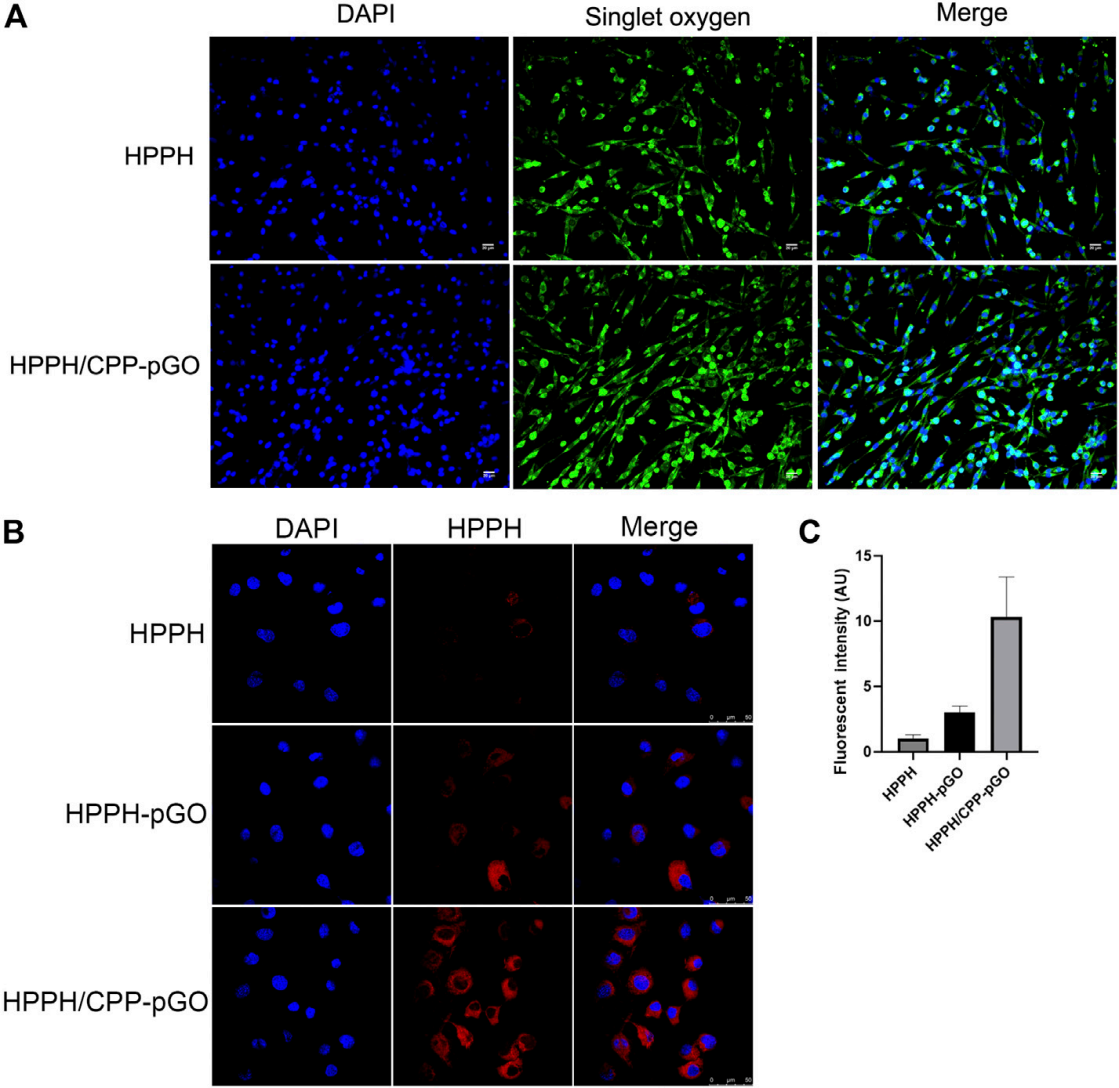
Cellular uptake of HPPH by MG-63 cells. Confocal images of MG-63 cells treated with HPPH, HPPH-pGO, or HPPH/CPP-pGO at 1 μM of HPPH.

The fluorescence intensity of SOSG revealed that the ^1^O_2_ generation of free HPPH was lower than that of HPPH/CPP-pGO (Figure 2A). Confocal imaging results showed that HPPH-pGO or HPPH/CPP-pGO treatment enhanced fluorescence intensity compared with free HPPH. Cells treated with HPPH/CPP-pGO exhibited the strongest fluorescence intensity (Figures 2B,C).

### Antiproliferation Effect of HPPH/EPI/CPP-pGO on MG-63 Cells

**FIGURE 3:**
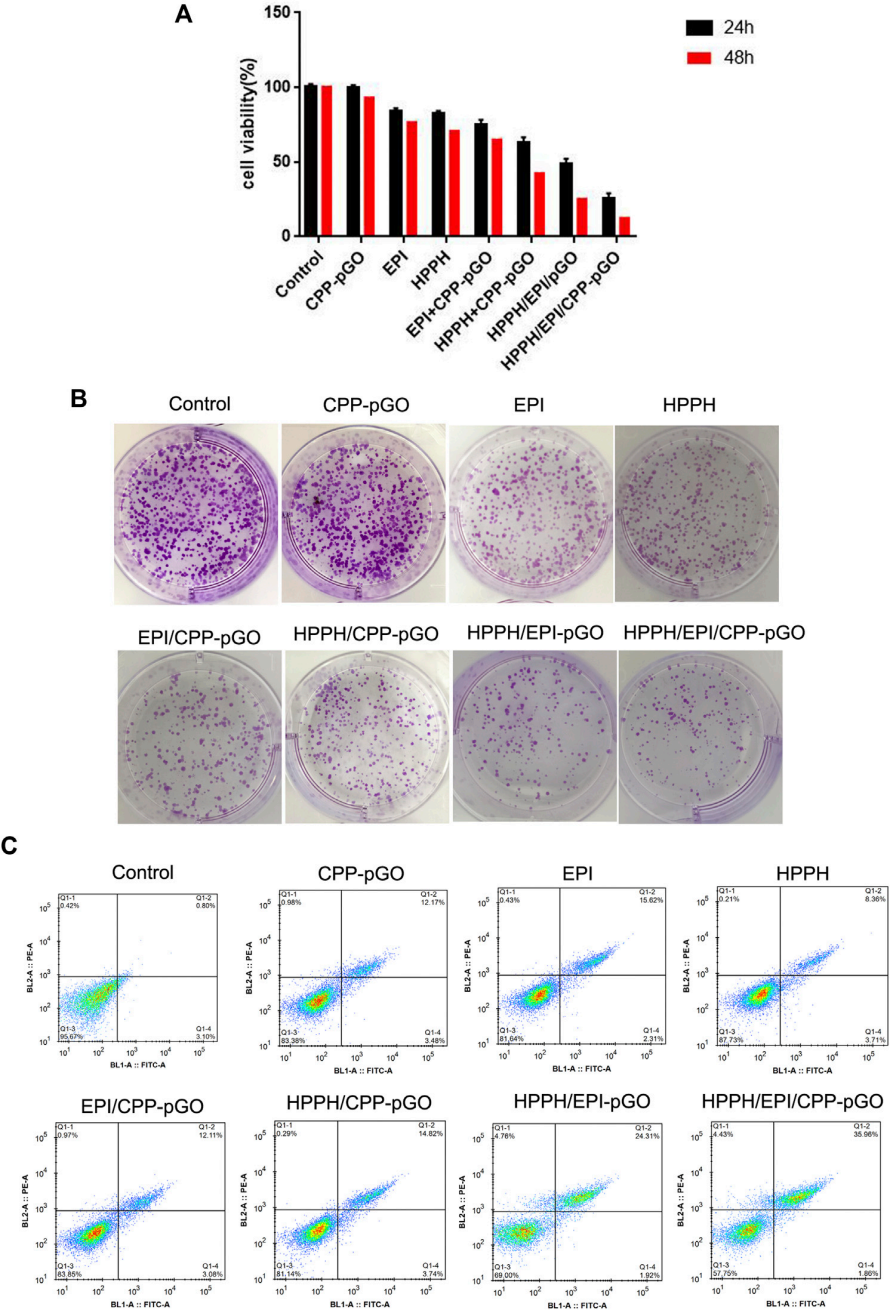
HPPH/EPI/CPP-pGO inhibited proliferation and colony formation of MG-63 cancer cells. **(A)** Cell viability of MG-63 cells treated with PBS (control), CPP-pGO, EPI (10 μg/mL), HPPH (1 μM), HPPH/CPP-pGO (containing 1 μM of HPPH), EPI/CPP-pGO (containing 10 μg/mL of EPI), HPPH/EPI/pGO (containing 1 μM of HPPH and 10 μg/mL of EPI), and HPPH/EPI/CPP-pGO (containing 1 μM of HPPH and 10 μg/ml of EPI), respectively, for 24 and 48 h by CCK-8 assay. **(B)** Colony forming number of MG-63 cells treated with PBS (control), CPP-pGO, EPI, HPPH, HPPH/CPP-pGO, EPI/CPP-pGO, HPPH/EPI/pGO, and HPPH/EPI/CPP-pGO, respectively, for 24 h by colony formation assay. **(C)** Cell apoptosis rate of U87 MG cells treated with PBS (control), CPP-pGO, EPI, HPPH, HPPH/CPP-pGO, EPI/CPP-pGO, HPPH/EPI/pGO, and HPPH/EPI/CPP-pGO, respectively, for 24 h by flow cytometry analysis.

CCK-8 assay found that, compared with control cells, CPP-pGO treatment had little effects on cell viability in MG-63 cells, indicating hypotoxicity of CPP-pGO on MG-63 cells (Figure 3A). In contrast, cell viability was obviously reduced after free EPI or HPPH treatment (Figure 3A) at 24 and 48 h. Compared with free EPI or HPPH treatment, MG-63 cells exhibited gradually decreasing cell viability after treatment with HPPH/EPI/CPP-pGO, followed by HPPH/EPI-pGO, HPPH/CPP-pGO, and EPI/CPP-pGO (Figure 3A). Consistently, colony formation results exhibited similar clone number in MG-63 cells treated with PBS and CPP-pGO, while the number of colonies was decreased after free EPI or HPPH treatment (Figure 3B). Compared with cells treated with free EPI or HPPH, the number of colony was reduced in cells with HPPH/EPI/CPP-pGO, followed by HPPH/EPI-pGO, HPPH/CPP-pGO, and EPI/CPP-pGO (Figure 3B). Moreover, flow cytometry analysis found that, compared with control cells, free EPI or HPPH treatment promoted the rate of apoptosis in MG-63 cells, while the percent of apoptotic cells was gradually reduced after MG-63 cells treatment with HPPH/EPI/CPP-pGO, followed by HPPH/EPI-pGO, HPPH/CPP-pGO, and EPI/CPP-pGO (Figure 3C).

### Effect of HPPH/EPI/CPP-pGO on Osteosarcoma Xenograft Mice

**FIGURE 4:**
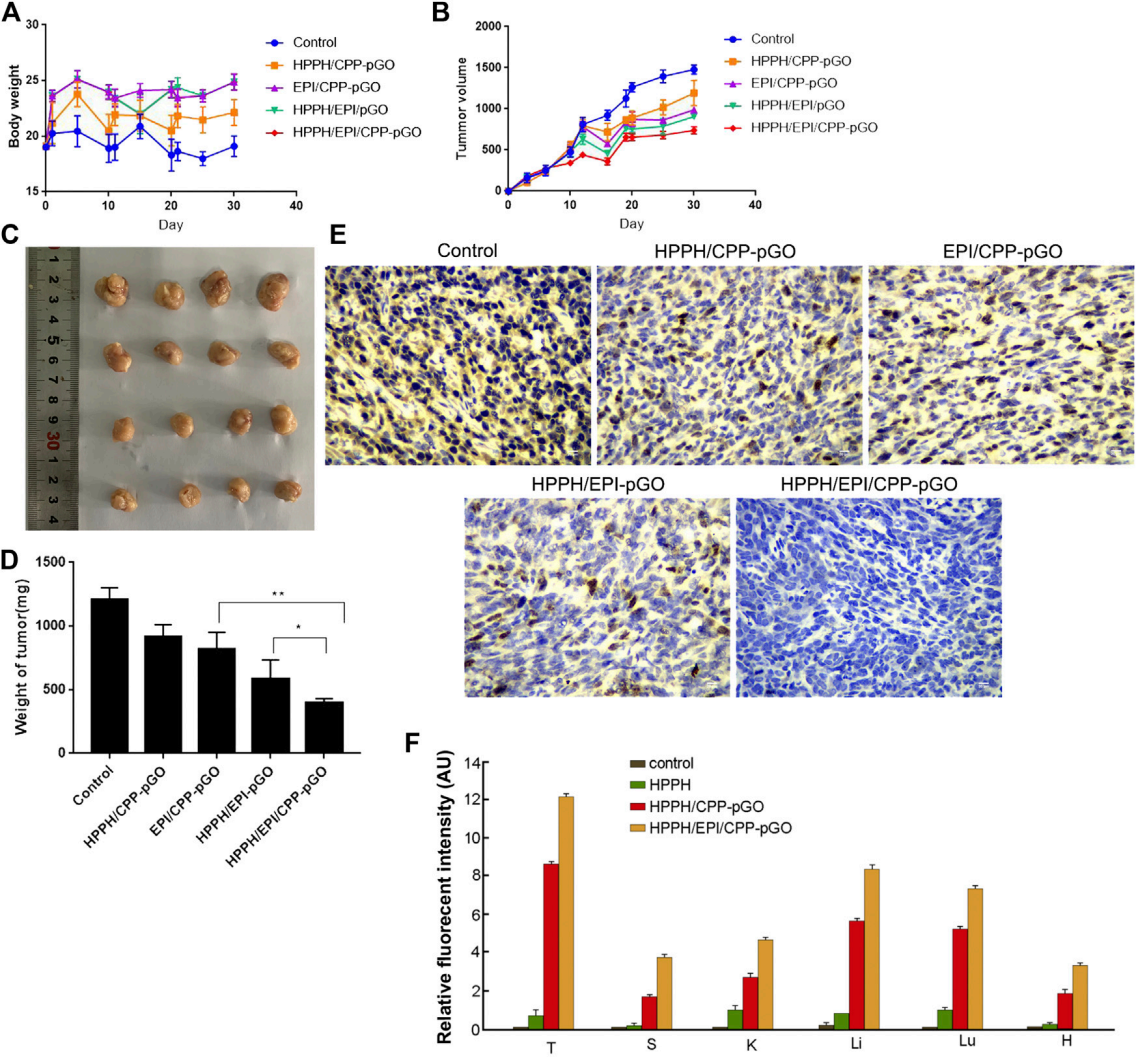
HPPH/EPI/CPP-pGO exhibited enhanced antitumor effect in osteosarcoma xenograft mice. **(A)** The body weight of osteosarcoma xenograft mice with different treatments, including saline solution (control group), HPPH/CPP-pGO (containing 1 mg/kg of HPPH), EPI/CPP-pGO (containing 5 mg/kg of EPI), HPPH/EPI/pGO (containing 1 mg/kg of HPPH and 5 mg/kg of EPI), or HPPH/EPI/CPP-pGO (containing 1 mg/kg of HPPH and 5 mg/kg of EPI). **(B)** The tumor volumes of mice with different treatments for 30 days. **(C)** The tumor image of mice with different treatments on day 30. **(D)** The tumor weights of mice with different treatments on day 30. **(E)** The Ki-67 expression of mice with different treatments by immunohistochemistry. **(F)** Quantitative analysis of the fluorescence intensity of the tumors and organs of mice after 24 h of drug injection. T, tumor; S, spleen; K, kidney; Li, liver; Lu, lung; H, heart. *N* = 4 in each group.

In our in vivo mouse model, the body weight of osteosarcoma xenograft mice was similar in control and treatment groups (Figure 4A). Moreover, we measured the blood biomarkers, including those of the liver and kidney toxicities, and found that they were not significantly changed by the treatment with the CPP-pGO, HPPH/EPI/CPP-pGO, or the PBS control (Supplementary Figure S1). These data suggested low toxicity due to potential nonspecific binding of CPP or pGO to normal tissues. Compared with control group, the tumor volume was reduced after HPPH/CPP-pGO or EPI/CPP-pGO treatment in a time-dependent manner, and mice in the HPPH/EPI/CPP-pGO group showed lower tumor volume than that in the HPPH/EPI-pGO group (Figures 4B,C). Furthermore, the tumor weight of mice was the lowest in the HPPH/EPI/CPP-pGO group, followed by HPPH/EPI-pGO, EPI/CPP-pGO, and HPPH/CPP-pGO groups (Figure 4D). Consistently, IHC showed that Ki67 expression trend in various groups was consistent with the tumor weight of mice (Figure 4E). The tumor tissues and major organs were collected 24 h after drug injection to study the biodistribution of the HPPH/CPP-pGO and free HPPH in the mouse (Figure 4F). We found strong HPPH fluorescence in the tumor sections of the HPPH/CPP-pGO- and HPPH/EPI/CPP-pGO-treated groups, but there were no obvious fluorescence signals in the control and the free HPPH treated group. These results suggested the enhanced antitumor effect of HPPH/EPI/CPP-pGO on osteosarcoma xenograft mice.

## Discussion

This study successfully prepared HPPH/EPI/CPP-pGO. The ^1^O_2_ generation and cellular uptake of HPPH were significantly increased after CPP and pGO modification compared with free HPPH. In addition, compared with control cells, CPP-pGO treatment has less effects on cell proliferation and apoptosis in MG-63 cells. However, compared with free HPPH or EPI, HPPH/EPI/CPP-pGO treatment significantly inhibited cell viability and clone number, as well as induced cell apoptosis, followed by HPPH/CPP-pGO or EPI/CPP-pGO, HPPH/EPI/CPP-pGO, and HPPH/EPI-pGO in MG-63 cells. Furthermore, the tumor volume and weight of osteosarcoma xenograft mice were obviously decreased after free HPPH or EPI treatment compared with control group, which were further reduced in other groups, especially in HPPH/EPI/CPP-pGO.

EPI has been widely reported to be applied into the treatment of breast cancer (Izgi et al., 2016), gastric cancer (Pan et al., 2018), and colorectal cancer (Cacan and Ozmen, 2020). PDT using irradiated HPPH had also been reported to be an effective therapeutic method for cancers (Chen et al., 2019; Shafirstein et al., 2016; Tracy et al., 2018). Consistently, this study showed that free EPI or free HPPH reduced cell viability, colony forming number, and induced cell apoptosis in MG-63 cells. However, the clinical application of various hydrophobic photosensitizers and anticancer drugs is limited due to poor water solubility. In view of the excellent biocompatibility and highly hydrophilic nature, GO has been considered as a promising drug delivery system to overcome hydrophobicity (Deb and Vimala, 2017; Lv et al., 2016). Previous study has reported that the release of gallic acid can be improved by gallic-acid-loaded GO and gallic-acid-loaded GO targeted to suppress cancer cell growth but not normal cells (Dorniani et al., 2016). A study on drug-resistant breast cancer also showed that compared with free adriamycin, adriamycin loaded GO exhibits enhanced ability to inhibit cell proliferation and induce apoptosis (Zhi et al., 2013). Herein, in order to improve drug delivery, EPI and/or HPPI were loaded into the pGO. Consistently, we found that, compared with free EPI or HPPI, both HPPH/CPP-pGO and EPI/CPP-pGO significantly diminished cell proliferation and induced cell apoptosis in MG-63 cells. Furthermore, this study revealed that the combination of EPI and irradiated HPPH exhibited preferable antitumor effect in MG-63 cells and osteosarcoma xenograft mice, which was consistent with previous clinical studies (Maria et al., 2018; Wentrup et al., 2016).

Furthermore, we focused on whether CPP modified pGO could further improve antitumor effects of EPI and irradiated HPPH. CPPs-dependent drug delivery system has been used for the treatment of neurological diseases, asthma, ischemia, diabetes, and cancers. Dubikovskaya et al. connected octa-arginine R8 with the anticancer drug paclitaxel through a disulfide bond to form the R8-paclitaxel covalent, and the results showed that the covalent of R8-paclitaxel was more likely to induce tumor cell apoptosis than paclitaxel alone in paclitaxel resistant tumor models (Dubikovskaya et al., 2008). In addition, in order to increase the cytotoxicity and targeted delivery of anticancer drugs, Lee et al. used chemical methods to combine doxorubicin, TAT and polymeric chitosan backbone to produce cell-penetrating chitosan/doxorubicin/TAT chimera. Compared with chimeric doxorubicin or chitosan/doxorubicin, this chimera showed more effective cell internalization and enhanced tumor localization, thereby significantly inhibiting tumor growth (Lee et al., 2011). Consistently, our study confirmed that HPPH/EPI/CPP-pGO treatment further inhibited cell growth *in vitro* and *in vivo* compared with HPPH/EPI-pGO. These results indicated that HPPH/EPI/CPP-pGO possessed enhanced antitumor effects.

In conclusion, this study successfully developed HPPH/EPI/CPP-pGO, which had superior osteosarcoma tumor-inhibiting effects *in vitro* and *in vivo*. Overall, HPPH/EPI/CPP-pGO might be a promising therapeutic nanomedicine for osteosarcoma targeting chemotherapy.

## Data Availability

The raw data supporting the conclusions of this article will be made available by the authors, without undue reservation.
