# Supplementary material for: Codelivery of Anticancer Drug and Photosensitizer by PEGylated Graphene Oxide and Cell Penetrating Peptide Enhanced Tumor-Suppressing Effect on Osteosarcoma
Source: Front Mol Biosci. 2021 Mar 31;7:618896. doi: 10.3389/fmolb.2020.618896 (PMC8060914; doi:10.3389/fmolb.2020.618896)
Supplement: Supplementary file 1 [file presentation1.pdf]

frontiers

For changes to the authorship of manuscripts, Frontiers requires that all authors complete and sign the 'Authorship Change' form. The affected author(s) must also sign this form as a way of consenting to the change(s) and agreeing to the new Author Contributions, Conflict of Interest and Acknowledgment sections.

In accordance with the ICMJE guidelines, when determining authorship the following criteria should be observed:

- Substantial contributions to the conception or design of the work; or the acquisition, analysis, or interpretation of data for the work; AND
- Drafting the work or revising it critically for important intellectual content; AND
- Final approval of the version to be published; AND
- Agreement to be accountable for all aspects of the work in ensuring that questionsrelated to the accuracy or integrity of any part of the work are appropriately investigatedand resolved.

Contributors who meet fewer than all 4 of the above criteria for authorship should not be listed as authors, but they should be acknowledged (as per the ICMJE guidelines).

If you have any questions please contact us.

Please note that we cannot proceed until this issue has been resolved.

Note that the form should be manually signed or contain valid e-signatures (an electronic version of a signature that can be uniquely identified and linked to the signatory, as shown in the example below). Typed names are not considered valid e-signatures.

- Digital stylus to sign the form can be used.
- The signatures can be on multiple versions of the form as long as the main information is filled in.
- If the signatures are on multiple files, you can zip these and upload them together.
- Useful link: https://helpx.adobe.com/acrobat/using/signing-pdfs.html

Should you provide a form with invalid signatures, we will not be able to consider your request for authorship change.

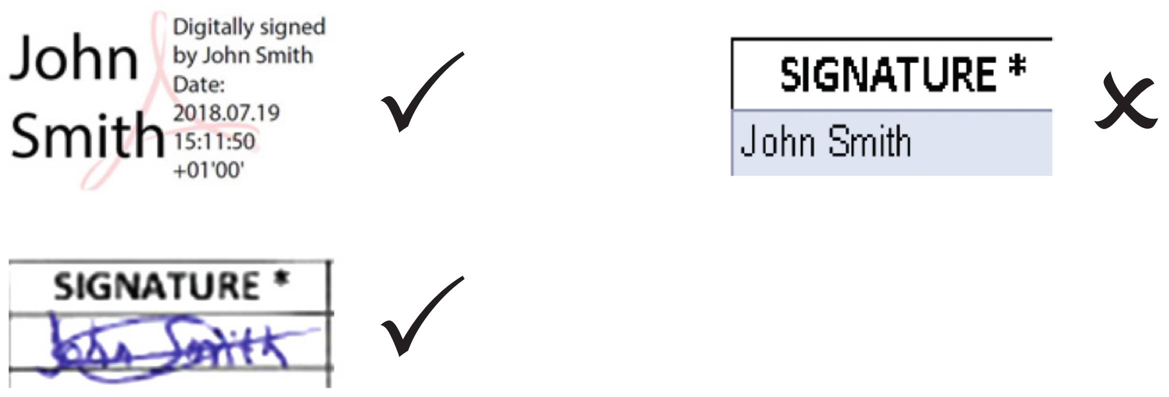

FORM FOR AUTHORSHIP CHANGE

Manuscript ID number: 618896

DOI:

(Please leave blank if not applicable)

Manuscript title: Co-delivery of anticancer drug and photosensitize by pegylated graphene oxide and cell p

Please tick as appropriate:

Addition of new author(s) ○ Addition and removal of author(s) ◉

Removal of author(s) ○ Change in the order of authors ○

Reason for authorship change:

For further study, due to the substantial contribution of Yifei Zhang, Yunfeng Wu, Taijin Lan, Shihong Su, Yao Chen in the revised manuscript during the revision stage, included saw the decision Letter, checklist, edited manuscript

and follow all editorial requirements, they also supported our work with their funding. They should be considered as co-author.
A few months before, Yibin Meng et al. looked like a success story in the face of the coronavirus pandemic. They not only stopped experiments in the lab, and continue to devote their time and energy to contain this pandemic. After communication, they decided not to sign the study. In a word, We are making this change because new authors meet

Details of author(s) to be added to the manuscript

First name(s)	Surname	Email address	Organization name (Department/University)	City/Country	Signature
Yifei	Zhang	yiouchao81085@163.c	Department of Human Anato	Chengdu	Yifei Zhang
Yunfeng	Wu	yunfen9586679288@12	Department of Orthopaedics,	Hefei	Yunfeng Wu
Taijin	Lan	jinthqt849621@163.cor	Department of Human Anato	Chengdu	Taijin Lan
Shihong	Su	shihuitunmei4216@126	Department of Respiratory an	Hefei	Shihong Su
Yao	Chen	yaofuyelu9752491@16	Department of Human Anato	Chengdu	Yao Chen

Details of author(s) to be removed from the manuscript

First name(s)	Surname	Email address	Organization name (Department/University)	City/Country	Signature
Xinliang	Zhang	huanbi88127383@126.	Honghui Hospital, Xi'an Jiaot	Xi'an	Xinliang Zhang
Wenjie	Gao	kekan8482339430@12	Sun Yat-sen Memorial Hospi	Guangzhou	Weijie Gao
Jijun	Chen	congzhan1055187@12	First Affiliated Hospital of Ai	Xi'an	Jijun Chen
Yunshan	Guo	luecou9254946@126.c	Honghui Hospital, Xi'an Jiaot	Xi'an	Yunshan Guo
(5) Jinwen	(5)Zhu	(5)dimu434880487@12	(5, 6)Honghui Hospital, Xi'an	(5,6)Xi'an	Jinwen Zhu

Yibin Meng

frontiers

Current Author Contribution Statement:

XLZ and WJG conceived the study and the figures. JJC and YSG collected the data. YBM wrote the manuscript and drew the figures. JWZ coordinated the study. All authors read and approved the final version of the manuscript.

New Author Contribution Statement:

(If the statement currently in the Manuscript/Proof is correct, please leave the below blank)

Yifei Zhang and Yunfeng Wu conceived the study and the figures. Taijin Lan and Shihong Su collected the data. Yao Chen wrote the manuscript and drew the figures. Yifei Zhang and Yao Chen coordinated the study. All authors read and approved the final version of the manuscript.

Current Conflict of Interest Statement:

All authors reported that they have no financial conflicts of interest.

New Conflict of Interest Statement:

(If the statement currently in the Manuscript/Proof is correct, please leave the below blank)

All authors reported that they have no financial conflicts of interest.

Current Acknowledgment Statement:

N/A

New Acknowledgment Statement:

(If the statement currently in the Manuscript/Proof is correct, please leave the below blank)

We are grateful to Dr.Yibin Meng et al. for help in improving this manuscript.

Please provide the final authors list in the order they should appear in the final publication

Note that the final spelling and presentation of Author names (special characters, middle names, etc) will be as provided in the Manuscript/Proof.

First name(s)	Surname	Email address	Signature	Date
Xinliang	Zhang	huanbi88127383@126.com	Xinliang Zhang	Dec 20, 2020
Wenjie	Gao	kekan8482339430@126.com	Wenjie Gao	Dec 20, 2020
Jijun	Chen	congzhan1055187@126.com	Jijun Chen	Dec 20, 2020
Yunshan	Guo	luecou9254946@126.com	Yunshan Guo	Dec 20, 2020
Jinwen	Zhu	dimu434880487@126.com	Jinwen Zhu	Dec 20, 2020
Yibin	Meng	quexia572263@126.com	Yibin Meng	Dec 20, 2020
Yifei	Zhang	yiouchao81085@163.com	Yifei Zhang	Dec 20, 2020
Yunfeng	Wu	yunfen9586679288@126.com	Yunfeng Wu	Dec 20, 2020
Taijin	Lan	jinthqt849621@163.com	Taijin Lan	Dec 20, 2020
Shihong	Su	shihuitunmei4216@126.com	Shihong Su	Dec 20, 2020
Yao	Chen	yaofuyelu9752491@163.com	Yao Chen	Dec 20, 2020

* By signing this form you agree **a**) for your name to added to/removed from the manuscript, **b**) to the new Author Contribution, Conflict of Interest and Acknowledgment statements and **c**) to the final author's list as listed in the form.

frontiers
